# Organofluorine chemistry: Difluoromethylene motifs spaced 1,3 to each other imparts facial polarity to a cyclohexane ring

**DOI:** 10.3762/bjoc.12.281

**Published:** 2016-12-22

**Authors:** Mathew J Jones, Ricardo Callejo, Alexandra M Z Slawin, Michael Bühl, David O'Hagan

**Affiliations:** 1 EaStCHEM School of Chemistry, University of St. Andrews, St. Andrews, Fife KY16 9ST, UK

**Keywords:** aliphatic rings, C–F bond, cyclohexane conformation, difluoromethylene group, organofluorine chemistry

## Abstract

2,2-Dimethyl-5-phenyl-1,1,3,3-tetrafluororocyclohexane has been prepared and characterised as an example of a facially polarised cyclohexane containing 1,3 related CF_2_ groups. The dipolar nature of the ring arises from the axial orientation of two of the C–F bonds pointing in the same direction, and set by the chair conformation of the cyclohexane. This electrostatic profile is revealed experimentally both in the solid-state (X-ray) packing of the rings and by solution (NMR) in different solvents. A computationally derived electrostatic profile of this compound is consistent with a more electronegative and a more electropositive face of the cyclohexane ring. This placing of CF_2_ groups 1,3 to each other in a cyclohexane ring is introduced as a new design strategy which could be applicable to the preparation of polar hydrophobic cyclohexane motifs.

## Introduction

Selective incorporation of fluorine atoms is a powerful strategy for modulating the properties of organic compounds [1–3]. For instance, the replacement of hydrogen by fluorine is commonly practised in medicinal [4–5] and agrochemical [6–7] research programmes. The dipole moment associated with the C–F bond has also rendered fluorine containing compounds important in the development of organic materials such as liquid crystals [8–9]. Strategic fluorination can add polarity to a molecule, however, such compounds do not generally increase in their hydrophilic capacity, thus selective fluorination leads to polar-hydrophobic properties [10]. Although selective mono-fluorination and CF_3_ incorporation have had wide currency, the difluoromethylene (CF_2_) motif has received relatively less attention despite possessing unique properties [11–14]. For example CH_2_F_2_ shows the highest molecular dipole (1.97 D) of the fluoromethane series, relative to CH_3_F (1.87 D) or CHF_3_ (1.65 D) [15–16]. Also (poly)vinylidine fluoride (~CF_2_CH_2_~) is the prototypical piezoelectric polymer, when the CF_2_ group are orientated (poled) in the melt [17].

**Figure 1:**
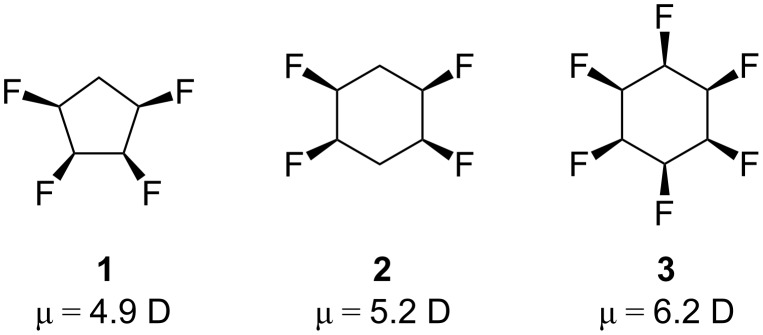
Selected fluorinated polar alicyclic scaffolds.

The search for new and more diverse polar hydrophobic scaffolds is attractive for drug discovery research and new materials [18]. In our own lab we have been exploring the synthesis and properties of differently fluorinated aliphatic structures based on the cyclopentane (**1**) and cyclohexane (**2** and **3**) rings, compounds which can show quite large dipole moments depending on the stereochemistry [19–20] (Figure 1). All-*syn* 1,2,3,4,5,6-hexafluorocyclohexane, which was prepared recently, emerges as among the most polar aliphatic compounds reported in the literature [21]. The specific arrangement with all of the fluorines “up” induces a facial polarisation to the cyclohexane, a unique phenomenon which may have unexplored utility in organic chemistry. For example derivatives of these compounds could be potentially useful as polar hydrophobic substituents in drug discovery that will simultaneously contact both electropositive and electronegative domains within a target protein.

**Scheme 1:**
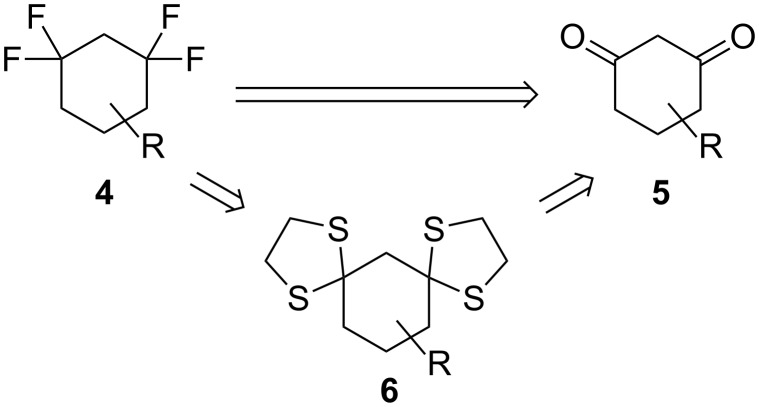
Retrosynthetic plan to the preparation of 1,1,3,3-tetrafluorocyclohexane structures.

In this paper, we now report the design, synthesis and characterisation of a novel tetrafluorocyclohexane structure **4**, rendered polar by placing two CF_2_ groups located 1,3 from each other (Scheme 1). This forces two C–F bonds to lie 1,3-diaxial to each other while the cyclohexane adopts the classic chair conformation. To the best of our knowledge, 2,2,6,6-tetrafluorocyclohexanol is the only derivative containing this feature reported so far. It was prepared in an eight step synthesis [22]. At the outset we explored a more direct synthesis of this arrangement based on deoxofluorination of 1,3-diketones **5** with DAST or Deoxo-Fluor^®^ [23] (Scheme 1). An alternative route envisaged a fluorodesulfurisation using 1,3-bis-dithianes **6** as substrates [24] (Scheme 1). Both synthetic approaches have been widely used for the introduction of the CF_2_ group in open-chain and (macro)cyclic systems [23–24], but not in a 1,3 relationship.

## Results and Discussion

**Scheme 2:**
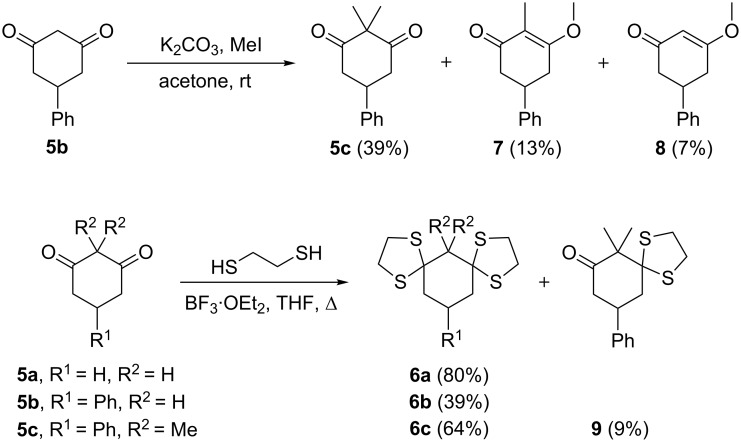
Preparation of starting materials **5c** and **6a–c**.

The preparation of diketones **5a–c** and bis-dithianes **6a–c** was carried out by previously described methods. The dimethyl-diketone **5c** was prepared by double methylation of **5b** [25] (Scheme 2). Two different *O*-alkylated products **7** and **8** were also obtained as significant byproducts.

The reaction of ketones **5a–c** with 1,2-ethanedithiol in the presence of catalytic BF_3_·OEt_2_ afforded the corresponding bis-dithianes **6a–c** in moderate to good yields [26] (Scheme 2). The reaction of diketone **5c** also afforded the monoderivatised ketone **9** as a minor product.

**Scheme 3:**
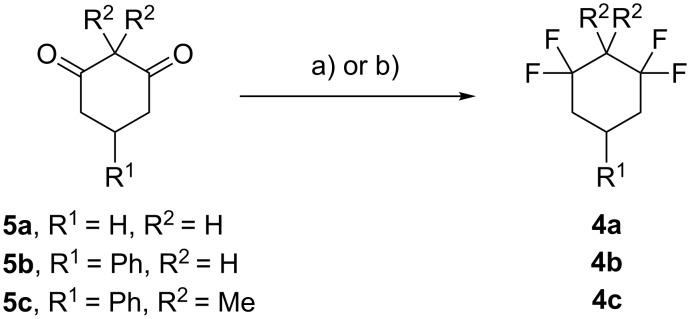
Deoxofluorination of diketones **5**. Reagents and conditions: a) DAST, DCM, rt, overnight, **4a** (traces), **4b** (traces), **4c** (14%); b) Deoxo-Fluor^®^, DCM, rt, overnight, **4a** (traces), **4b** (traces), **4c** (10%).

Direct fluorination of diketones **5a–c** was explored with DAST or Deoxo-Fluor^®^ (Scheme 3). When diketones **5a** and **5b** were treated with DAST or Deoxo-Fluor^®^, either neat or in DCM as a solvent, only complex and intractable products were obtained. These highly enolisable diketones [27–28] were incompatible with deoxyfluorination. In order to block enolisation, diketone **5c** containing two methyl groups α to the ketones was subject to fluorination with DAST and Deoxo-Fluor^®^. In this case the reaction was successful and the desired tetrafluorocyclohexane **4c** could be obtained, most preferably with DAST although in a low overall yield (Scheme 3). The presence of the *gem*-dimethyl motif renders the carbonyl groups independent of each other and this facilitates the difluorination process relative to **5a** and **5b**. Nevertheless, other unidentified byproducts were also obtained, which made purification of **4c** by column chromatography difficult and compromised the yield.

**Scheme 4:**
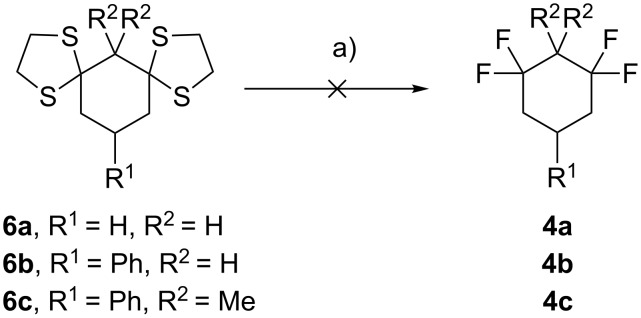
Fluorodesulfurisation of bis-dithianes **6**. Reagents and conditions: a) NIS, HF·Py, DCM, −78 °C to rt, overnight.

Treatment of bis-dithianes **6** with NIS and HF·Py as previously described for linear systems [28] proved unsuccessful (Scheme 4) and did not offer a way forward.

**Figure 2:**
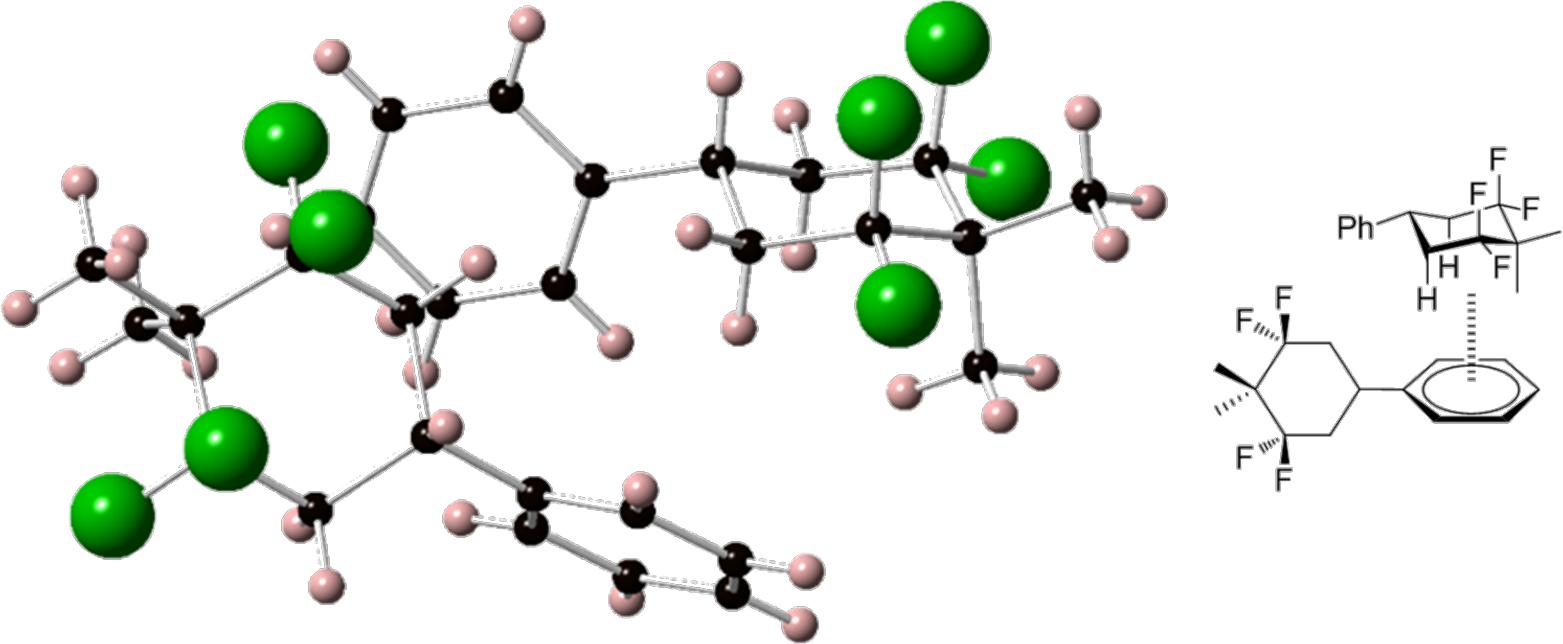
X-ray structure of compound **4c**. The image shows two molecules stacked with the non-fluorine face pointing to an adjacent phenyl ring consistent with an electrostatic ordering. The structure has been deposited in the Cambridge Crystallographic Data Centre (CCDC) 1502954.

Tetrafluorocyclohexane **4c** was a crystalline solid (mp 70 ºC). The structure of this compound was established by NMR, showing a characteristic AB system in ^19^F NMR for axial/equatorial fluorines, and a triplet (^1^*J_CF_* = 250 Hz) in ^13^C NMR for the CF_2_ group. In addition, single crystal X-ray diffraction data were obtained for **4c** which confirmed the structure (Figure 2). The C–CF_2_–C angles in the cyclohexane ring are considerably wider (≈116°) than that of the C–CH_2_–C angles (≈112°), which is now recognised as a general phenomenon of the CF_2_ group in an aliphatic chain/ring [10–13]. The two axial fluorines flex away from each other with widened right angles of 96.3°, distorting a co-parallel orientation. This distortion is also observed for the 1,3-diaxial C–F bonds in the solid state structures of **2** [20] and **3** [21] consistent with electrostatic dipolar repulsion between these C–F bonds and indicative of the origin of the polarity of these molecules. In terms of intermolecular packing in the solid state there are interactions between the non-fluorine face of the cyclohexyl ring and the phenyl group of a neighbouring molecule (Figure 2). These C-H···π interactions are a feature of the solid state packing found in phenyl derivatives of **2** [29].

**Figure 3:**
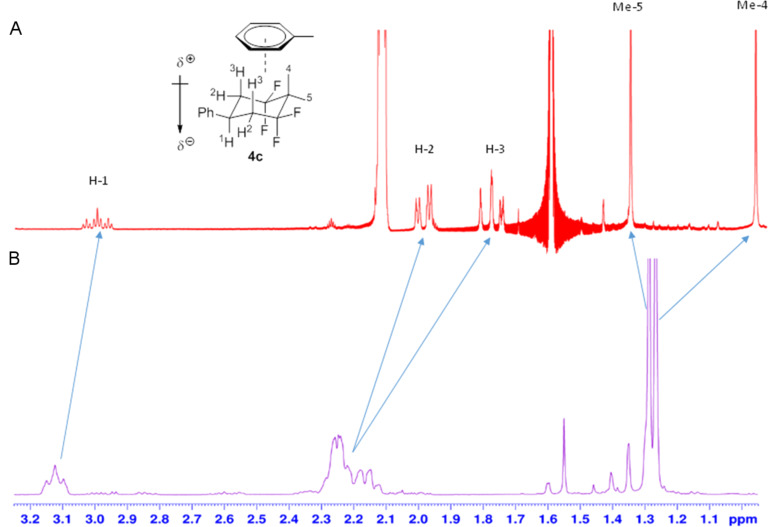
^1^H NMR spectra of **4c**. A) shows the spectrum in [^2^H_8_]-toluene, and B) shows the spectrum in chloroform (CDCl_3_). There are significant upfield-shifted proton signals in toluene, consistent with an electrostatic interaction between toluene and the electropositive hydrogen face of 1,1,3,3-tetrafluorocyclohexyl **4c**.

This electrostatic ordering was reinforced by an NMR shift experiment in different solvents. Selected ^1^H NMR signals of **4c** showed a very clear upfield shift in [^2^H_8_]-toluene when compared with the spectrum obtained in chloroform (CDCl_3_) (Figure 3). In particular, the two axial protons H-3 and the axial methyl group at C-4 display higher upfield shifts in toluene (Δδ ≈ 0.5 ppm and Δδ ≈ 0.3 ppm, respectively), whereas that of the equatorial protons H-2, the equatorial methyl group at C-5 and the axial proton H-1 do not experience shifts of the same magnitude (Δδ ≈ 0.2 ppm, Δδ ≈ −0.1 ppm and Δδ ≈ 0.1 ppm, respectively). These observations are consistent with the anisotropic influence of the electronegative aromatic solvent (toluene) interacting with the more electropositive (non-fluorine) face of the cyclohexane ring (Figure 3).

**Figure 4:**
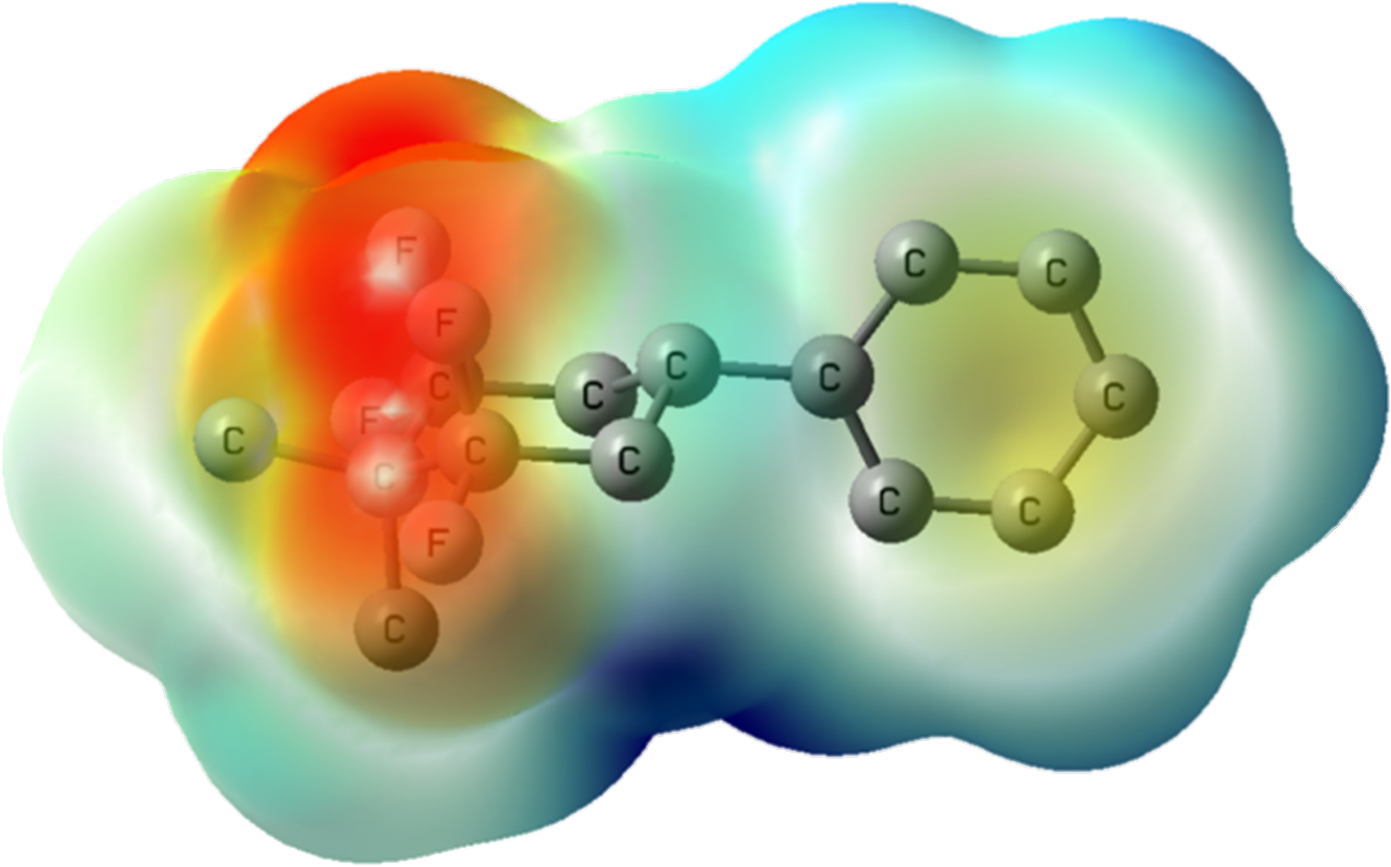
Electrostatic potential map for **4c** calculated at the B3LYP/6-311G(d,p) level for an optimised structure calculated at the B3LYP-D3/6-311+G(d,p) level. The image is plotted on a colour scale from −0.003 a.u. (red) to +0.003 a.u. (blue) and mapped onto an isodensity surface (ρ = 4 × 10^−4^ a.u.); hydrogen atoms are omitted for clarity.

To extend these experimental observations, a DFT computational study, at the B3LYP-D3/6-311+G(d,p) level, was carried out in order to obtain an electrostatic potential map of cyclohexane **4c**. The profile confirms the presence of an electronegative face (in red) determined by the two 1,3-diaxial C–F bonds and an electropositive face (dark blue), which is most intense at the hydrogens of the diaxial C–H bonds pointing antiperiplanar to the diaxial C–F bonds (Figure 4). A molecular dipole moment for compound **4c** was calculated at 3.1 D and can be compared for example to 1,2,4,5-tetrafluorocyclohexane (**2**, 5.2 D) [18].

## Conclusion

In this study we have prepared a novel polar aliphatic structure **4c** based on the 1,1,3,3-tetrafluorocyclohexyl motif. The compound was prepared by direct difluorination of the corresponding α,α-dimethylated 1,3-diketone with DAST. The presence of the two 1,3-diaxial fluorine atoms in structure **4c** is responsible for the facial polarisation on the cyclohexane ring. We have demonstrated the intermolecular electrostatic interaction of the positive non-fluorous face with the π-system of an aromatic ring by single crystal X-ray diffraction and NMR experiments. Additionally, the polarity of **4c** has been calculated by computational optimisations. It follows that functionalised cyclohexanes containing the 1,3-di-CF_2_ motif would offer new possibilities for the design of performance molecules extending from organic materials research to medicinal chemistry.

## Supporting Information

File 1 Experimental part and NMR spectra of new synthesised compounds.
