# Supplementary material for: Organofluorine chemistry: Difluoromethylene motifs spaced 1,3 to each other imparts facial polarity to a cyclohexane ring
Source: Beilstein J Org Chem. 2016 Dec 22;12:2823–7. doi: 10.3762/bjoc.12.281 (PMC5238547; doi:10.3762/bjoc.12.281)
Supplement: File 1 — Experimental part and NMR spectra of new synthesised compounds. [file Beilstein_J_Org_Chem-12-2823-s001.pdf]

Supporting Information

for

Organofluorine chemistry: Difluoromethylene motifs spaced 1,3 to each other imparts facial polarity to a cyclohexane ring

Mathew J. Jones, Ricardo Callejo, Alexandra M. Z. Slawin, Michael Bühl and David O'Hagan*

Address: *EaStCHEM* School of Chemistry, University of St. Andrews, St. Andrews, Fife KY16 9ST, UK

Email: David O'Hagan – do1@st-andrews.ac.uk

* Corresponding author

Experimental part and NMR spectra of new synthesised compounds

Contents

1. General Information

2. Experimental procedures and characterisation

3. NMR spectra of novel compounds

1. General Information

Chemicals and solvents were acquired from Sigma-Aldrich, Fisher Chemical or VMR Chemicals. Specifically, DAST was purchased from Flurochem and Deoxo-Fluor®, dione **5a** and dione **5b** were purchased from Sigma-Aldrich.

Thin layer chromatography used Merck silica gel 60 F_{254} aluminium-backed TLC plates and were visualised under UV light (254 nm) or by thermal development after dipping in either a) ethanolic phosphomolybdic acid solution or basic potassium permanganate solution. **Column chromatography** used Fluka Analytical silica gel 60 230-400 mesh obtained from Sigma-Aldrich, run with solvents as supplied under a stream of compressed air.

Melting points were determined using an Electrothermal melting point apparatus and are uncorrected.

NMR spectra were recorded on a Bruker Avance 400 spectrometer or Bruker Avance II 400 spectrometer (^{1}H at 400 MHz, ^{13}C at 100 MHz, ^{19}F at 376 MHz), Bruker Avance III 500 spectrometer or Bruker Avance III HD 500 spectrometer (^{1}H at 500 MHz, ^{13}C at 125 MHz, ^{19}F at 470 MHz). All chemical shifts (δ) are reported in parts per million (ppm) relative to TMS $\delta<0.00$ ppm and were reported to the residual solvent signal. Spectra were analysed using TopSpin3.2. Samples were dissolved in deuterated solvents specified. Coupling constants (*J*) are given in Hertz (Hz) and are reported as an average to the nearest 0.1 Hz. Signal splitting are described as: s – singlet, d – doublet, t – triplet, dd – doublet of doublets, tt – triplet of triplets, m – multiplet.

Mass spectrometry was conducted at the University of St Andrews by Mrs. C. Horsburgh in the MS facility. Data was obtained by electrospray ionisation (ESI) on a Thermo Excalibur Orbitrap spectrometer or chemical ionisation (CI) on Micromass GCT spectrometer with methane as carrier gas. Additional data was obtained from the EPSRC National Mass Spectrometry Service Centre, Swansea; the APCI (atmospheric pressure CI) spectra were obtained on Xevo G2-S spectrometer using an ASAP solids probe.

The X-ray crystal structure was data was obtained on a Rigaku XtaLAB P200 diffractometer using multi-layer mirror monochromated Mo-Ka radiation and were analysed using CrystalMaker.

2. Experimental procedures and characterisation

Preparation of ketone 5c

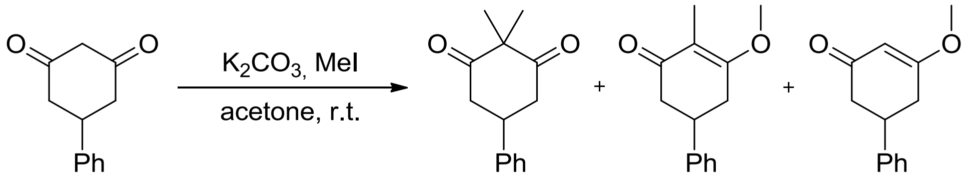

5b **5c** (39%) **7** (13%) **8** (7%)

K_2CO_3 (8.45 g, 61.1 mmol, 2.3 equiv.) and MeI (3.64 mL, 58.5 mmol, 2.2 eq) were added to a stirred solution of dione **5b** (5 g, 26.6 mmol, 1.0 equiv.) in acetone (84 mL). The mixture was heated under reflux overnight before being allowed to cool to room temperature. The mixture was diluted with DCM (100 mL) and the precipitate was removed by filtration and the filtrate was concentrated *in vacuo*. The residue was reconstituted with chloroform (100 mL) and concentrated. The residue was diluted with DCM (100 mL) and washed with water (100 mL). The aqueous phase was extracted with DCM (2 x 50 mL), dried over $MgSO_4$ and concentrated affording a crude yellow oil, which was purified by column chromatography, eluting with hexane and ethyl acetate (5:1), to give 2,2-dimethyl-5-phenyl-cyclohexanedione **5c** (2.24 g, 39%), 3-methoxy-2-methyl-5-phenylcyclohexen-2-one **7** (0.75 g, 13%) and 3-methoxy-5-phenylcyclohexen-2-one **8** (0.40 g, 7%).

Compound **5c**. White solid; **m.p.** 67-70 ^{o}C; **^{1}H NMR** (500 MHz, $CDCl_3$) δ_H 7.39-7.34 (2H, m, 2 CH_{Ar}), 7.30-7.26 (1H, m, CH_{Ar}), 7.23-7.19 (2H, m, 2 CH_{Ar}), 3.24-3.17 (1H, m, CH), 3.07-2.98 (2H, m, 2 $C\mathit{H}_{ax}H_{eq}$), 2.89 (2H, dd, *J* = 15.2, 4.6 Hz, 2 $CH_{ax}\mathit{H}_{eq}$), 1.43 (3H, s, Me), 1.33 (3H, s, Me); **^{13}C NMR** (125 MHz, $CDCl_3$) δ_C 209.7 (2 C=O), 141.8 (C_{Ar}), 129.2 (2 CH_{Ar}), 127.6 (2 CH_{Ar}), 126.6 (CH_{Ar}), 61.0 (*C*Me_2), 45.0 (2 CH_2), 36.0 (CH), 24.9 (Me), 19.9 (Me); **HRMS** (ESI^+) *m/z* calcd for $C_{14}H_{17}O_2$ $[M+H]^+$: 217.1223, found: 217.1219.

Compound **7**. White solid; **m.p.** 65-66 ^{o}C; **^{1}H NMR** (500 MHz, $CDCl_3$) δ_H 7.37 (2H, t, *J* = 7.4 Hz, 2 CH_{Ar}), 7.31-7.27 (2H, m, 2 CH_{Ar}), 7.23-7.19 (1H, m, CH_{Ar}), 3.82 (3H, s, MeO), 3.36-3.26 (1H, m, CH), 2.93-2.54 (4H, m, 2 CH_2), 1.74 (3H, s, Me); **^{13}C NMR** (125 MHz, $CDCl_3$) δ_C 197.9 (C=O), 170.8 (OC=), 143.1 (C_{Ar}), 129.0 (2 CH_{Ar}), 127.3 (CH_{Ar}), 127.9 (2 CH_{Ar}), 114.9 (C=), 55.5 (MeO), 43.2 (CH_2), 39.5 (CH), 33.1 (CH_2), 7.5 (Me); **HRMS** (ESI^+) *m/z* calcd for $C_{14}H_{17}O_2$ $[M+H]^+$: 217.1223, found: 217.1229.

Compound **8**. Yellow oil; **^{1}H NMR** (500 MHz, $CDCl_3$) δ_H 7.33 (2H, t, *J* = 7.6 Hz, 2 CH_{Ar}), 7.27-7.22 (3H, m, 3 CH_{Ar}), 5.44 (1H, s, HC=), 3.71 (3H, s, MeO), 3.39-3.31 (1H, m, CH), 2.71-2.53 (4H, m, 2 CH_2); **^{13}C NMR** (125 MHz, $CDCl_3$) δ_C 198.7 (C=O), 177.7 (OC=), 142.7 (C_{Ar}), 128.8 (2 CH_{Ar}), 127.1 (CH_{Ar}), 126.7 (2 CH_{Ar}), 102.2 (HC=), 55.9 (MeO), 43.9 (CH_2), 39.4 (CH), 36.5 (CH_2); **HRMS** (ESI^+) *m/z* calcd for $C_{13}H_{15}O_2$ $[M+H]^+$: 203.1067, found: 203.1062.

Preparation of bis-dithianes 6

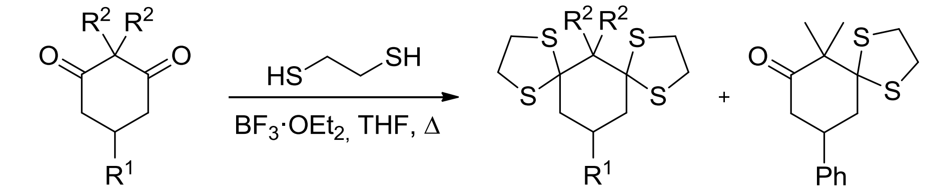

5a, R^1=H, R^2=H — **6a** (80%)
5b, R^1=Ph, R^2=H — **6b** (39%)
5c, R^1=Ph, R^2=Me — **6c** (64%) — **9** (9%)

Compound **6a**. Boron trifluoride etherate (5.5 mL, 44.6 mmol, 2 equiv.) and 1,2-ethanedithiol (3.74 mL, 44.6 mmol, 2 equiv.) was added to a stirred solution of dione **5a** (2.5 g, 22.3 mmol, 1 equiv.) in THF (60 mL) at room temperature. The mixture was heated under reflux with stirring for 24 hours. The solution was diluted with hot water (250 mL) affording a white crystalline precipitate, which was isolated by filtration. Recrystallisation of the residue from isopropyl ether afforded dithiane **6a** (4.61 g, 80%) as a yellow solid; **m.p.** 153-155 ^{o}C; **^{1}H NMR** (400 MHz, $CDCl_3$) δ_H 3.36-3.23 (8H, m, 4 CH_2S), 2.73 (2H, s, CH_2), 2.01-1.97 (4H, m, 2 CH_2), 1.85-1.79 (2H, m, CH_2); **^{13}C NMR** (125 MHz, $CDCl_3$): δ_C 67.9 (CS_2), 54.5 (CH_2), 42.6 (2 CH_2), 39.0 (4 CH_2S), 25.6 (CH_2). **HRMS** (ESI^+) *m/z* calcd for $C_{10}H_{16}S_4Na$ $[M+Na]^+$: 287.0027, found: 287.0020.

Compound **6b**. Boron trifluoride etherate (2.8 mL, 22.4 mmol, 2 equiv.) and 1,2-ethanedithiol (1.9 mL, 22.4 mmol, 2 equiv.) was added to a stirred solution of dione **5b** (2.11 g, 11.2 mmol, 1 equiv.) in THF (60 mL) at room temperature. The mixture was heated under reflux with stirring for 24 hours. The solution was washed with saturated sodium hydrogen carbonate solution (2 x 100 mL), saturated sodium hydroxide solution (2 x 100 mL), brine (100 mL) and dried over anhydrous $MgSO_4$. The solvent was evaporated *in vacuo* and the crude was purified by silica gel

chromatography, eluting with hexane and ethyl acetate (10:1), to give bis-dithiane **6b** (1.48 g, 39%) as a yellow solid; **m.p.** 180-182 °C; **^{1}H NMR** (500 MHz, $CDCl_3$) δ_H 7.33-7.28 (2H, m, 2 CH_{Ar}), 7.23-7.17(3H, m, 3 CH_{Ar}), 3.43-3.25 (8H, m, CH_2S), 3.15 (1H, tt, J = 12.4, 6.4 Hz, CH), 2.81-2.79 (2H, m, CH_2), 2.38-2.33 (2H, m, 2 $CH_{ax}H_{eq}$), 2.05 (2H, dd, J = 13.8, 12.5 Hz, 2 $CH_{ax}H_{eq}$); **^{13}C NMR** (125 MHz, $CDCl_3$): δ_C 144.6 (C_{Ar}), 128.7 (2 CH_{Ar}), 127.2 (2 CH_{Ar}), 126.7 (CH_{Ar}), 67.8 (CS_2), 53.8 (CH_2), 50.1 (2 CH_2), 43.1 (CH), 40.2 (2 CH_2S), 37.9 (2 CH_2S); **HRMS** (ESI^+) *m/z* calcd for $C_{16}H_{21}S_4$ $[M+H]^+$: 341.0521, found: 341.0518.

Compounds **6c** and **9**. Boron trifluoride etherate (1.66 mL, 13.5 mmol, 3 equiv.) and 1,2-ethanedithiol (1.13 mL, 13.5 mmol, 3 equiv.) was added to a stirred solution of dione **5c** (629 mg, 4.5 mmol, 1 equiv.) in THF (23 mL) at room temperature. The mixture was heated under reflux with stirring for 2 days. The solution was washed with saturated sodium hydrogen carbonate solution (2 x 100 mL), saturated sodium hydroxide solution (2 x 100 mL), brine (100 mL) and dried over anhydrous $MgSO_4$. The solvent was evaporated *in vacuo* and the crude was purified by silica gel chromatography, eluting with hexane and ethyl acetate (10:1), to give bis-dithiane **6c** (1.06 g, 64%) and the monoprotected dithiane **9** (0.15 g, 9%).

Compound **6c**. Colourless solid; **m.p**. 149-151 ºC; **^{1}H NMR** (500 MHz, $CDCl_3$) δ_H 7.33-7.28 (2H, m, 2 CH_{Ar}), 7.26-7.23 (2H, m, 2 CH_{Ar}), 7.22-7.18 (1H, m, CH_{Ar}), 3.36-3.21 (8H, m, 4 CH_2S), 3.21-3.16 (1H, m, CH), 2.58 (2H, t, J = 13.1 Hz, 2 $CH_{ax}H_{eq}$), 2.25 (2H, dd, J = 13.8, 3.5 Hz, 2 $CH_{ax}H_{eq}$), 1.63 (3H, s, Me), 1.60 (3H, s, Me); **^{13}C NMR** (125 MHz, $CDCl_3$) δ_C 144.6 (C_{Ar}), 128.7 (2 CH_{Ar}), 127.3 (2 CH_{Ar}), 126.6 (CH_{Ar}), 80.0 (2 CS_2), 48.4 (2 CH_2), 42.0 (2 CMe_2), 41.1 (2 CH_2S), 38.2 (CH), 28.1 (Me), 22.7 (Me); **HRMS** (ESI^+) *m/z* calcd for $C_{18}H_{25}S_4$ $[M+H]^+$: 369.0834, found: 369.0842.

Compound **9**. Colourless solid; **m.p**. 119-121 ºC; **^{1}H NMR** (500 MHz, $CDCl_3$) δ_H 7.34 (2H, t, J = 7.7 Hz, 2 CH_{Ar}), 7.28-7.22 (3H, m, 3 CH_{Ar}), 3.35-3.22 (5H, m, 2 CH_2S, CH), 2.93 (1H, t, J = 13.3 Hz, CH_{ax} $\alpha_{C=O}$), 2.88 (1H, t, J = 13.9 Hz, CH_{ax}), 2.56-2.50 (1H, m, CH_{eq}), 2.44-2.38 (1H, m, CH_{eq} $\alpha_{C=O}$), 1.46 (3H, s, Me), 1.38 (3H, s, Me); **^{13}C NMR** (125 MHz, $CDCl_3$) δ_C 211.2 (C=O), 143.1 (C_{Ar}), 128.8 (2 CH_{Ar}), 127.0 (CH_{Ar}), 126.7 (2 CH_{Ar}), 77.8 (CS_2), 55.0 (CMe_2), 47.4 (CH_2 $\alpha_{C=O}$), 43.9 (CH_2), 41.7 (CH), 40.7 (CH_2S), 39.3 (CH_2S), 25.8 (Me), 20.3 (Me); **HRMS** (ESI^+) *m/z* calcd for $C_{16}H_{20}OS_2$ $[M+Na]^+$: 315.0853, found: 315.0842.

Difluorination of diketones 5

In a Teflon® flask, DAST (1.89 mL, 14.3 mmol, 4 equiv.) or Deoxo-Fluor® (50% soln. in THF, 2.55 mL, 12.1 mmol, 4 equiv.) in DCM (10 mL) was added to a solution of dione **5c** (0.5 g, 3.57 mmol, 1 equiv.) in DCM (5 mL) and stirred overnight. The reaction was quenched by addition of the mixture to a stirred solution of DCM (100 mL) and saturated sodium hydrogen carbonate solution (100 mL) at 0 ^{o}C. The aqueous layer was separated and extracted with DCM (3 x 50 mL). The organic layer was washed with brine (100 mL), dried over magnesium sulfate and the solvent was evaporated *in vacuo*. Purification by several silica gel chromatography columns, eluting with hexane and ethyl acetate (30:1 to 20:1), afforded compound **4c** (0.13 g, 14%, from DAST; 0.09 g, 10%, from Deoxo-Fluor®) as a colourless crystalline solid; **m.p.** 69-71 ^{o}C; **^{1}H NMR** (500 MHz, $CDCl_3$) δ_H 7.34-7.28 (2H, m, 2 CH_{Ar}), 7.25-7.21 (1H, m, CH_{Ar}), 7.20-7.15 (2H, m, 2 CH_{Ar}), 3.12-3.08 (1H, m, CH), 2.26-2.17 (2H, m, 2 $CH_{ax}\mathit{H_{eq}}$), 2.16-2.06 (4H, m, 2 $C\mathit{H_{ax}}H_{eq}$), 1.27 (3H, s, Me), 1.25 (3H, s, Me); **^{13}C NMR** (125 MHz, $CDCl_3$) δ_C 141.6 (C_{Ar}), 129.2 (2 CH_{Ar}), 127.3 (CH_{Ar}), 126.8 (2 CH_{Ar}), 124.1 (t, *J* = 249 Hz, CF_2), 45.6 (*C*Me_2), 37.0 (2 CH_2), 34.6 (CH), 20.3 (Me), 12.5 (Me); **^{19}F NMR** (470 MHz, $CDCl_3$) δ_F −103.1−104.6 (4F, m, 2 CF_2); **HRMS** (APCI) *m/z* calcd for $C_{14}H_{17}F_4$ $[M+H]^+$: 261.1261, found: 261.1222.

Difluorination of bis-dithianes 6

In a Teflon® flask, HF·Py (1.3 mL, 71.7 mmol, 120 equiv.) was added to a solution of *N*-iodosuccinimide (1.08 g, 4.78 mmol, 8 equiv.) in DCM (9.5 mL) and stirred at −78 ^{o}C for 10 min. Then, a solution of dithiane **6c** (0.22 g, 0.6 mmol, 1 equiv.) in DCM (7.3 mL) was added dropwise to the above mixture over 10 min. The solution was then stirred at −78 ^{o}C for 4 h further before warming to room temperature overnight. The reaction was quenched by addition of the mixture to a stirred solution of DCM (100 mL) and saturated sodium bicarbonate solution (100 mL) at 0 ^{o}C. The aqueous layer was separated and extracted with DCM (3 x 50 mL). The organic layer was washed with saturated sodium thiosulfate solution (100 mL), brine (100 mL), saturated copper sulfate solution (100 mL) and dried over anhydrous $MgSO_4$. The solvent was evaporated *in vacuo* affording the corresponding crude.

3. NMR spectra of novel compounds

2,2-dimethyl-5-phenylcyclohexane-1,3-dione 5c

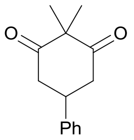

5c

^{1}H NMR

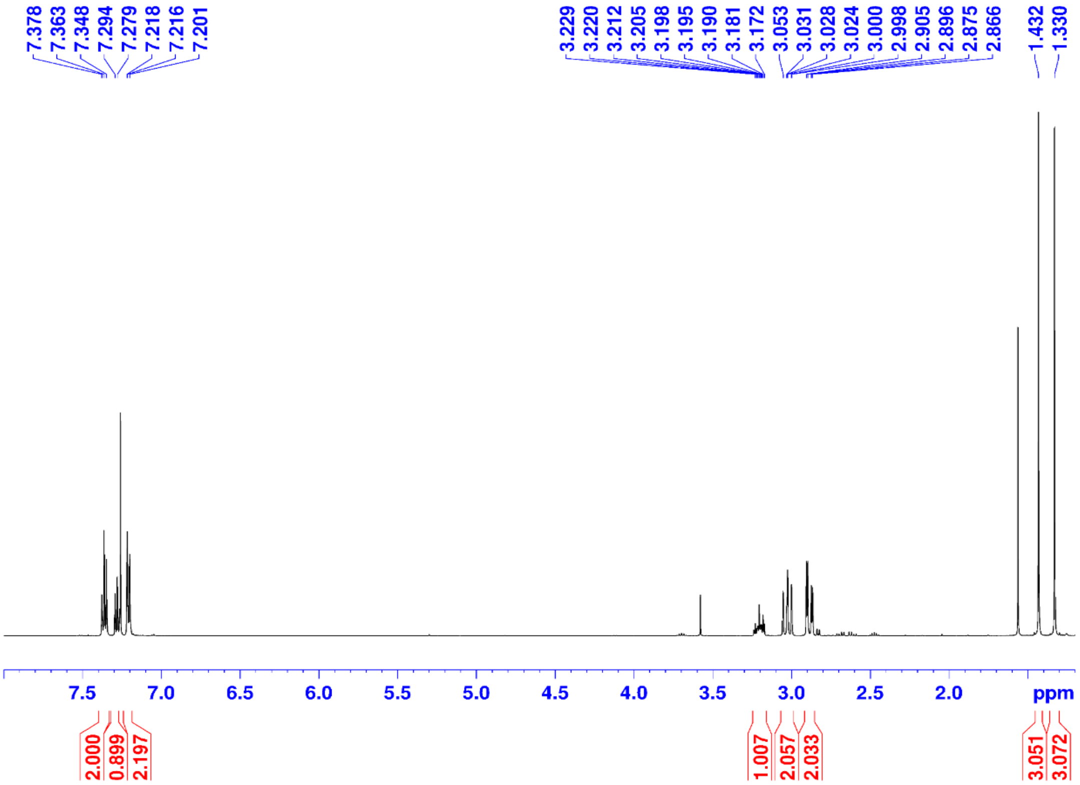

^{13}C NMR

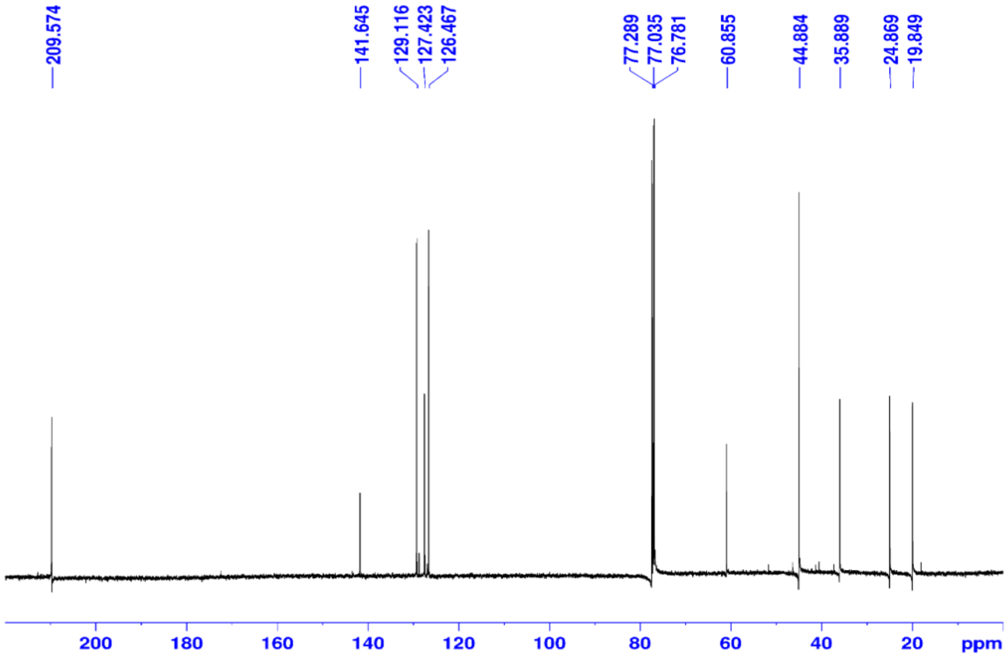

3-methoxy-2-methyl-5-phenylcyclohex-2-ene-1-one 7

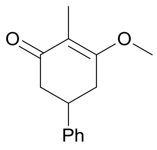

7

^{1}H NMR

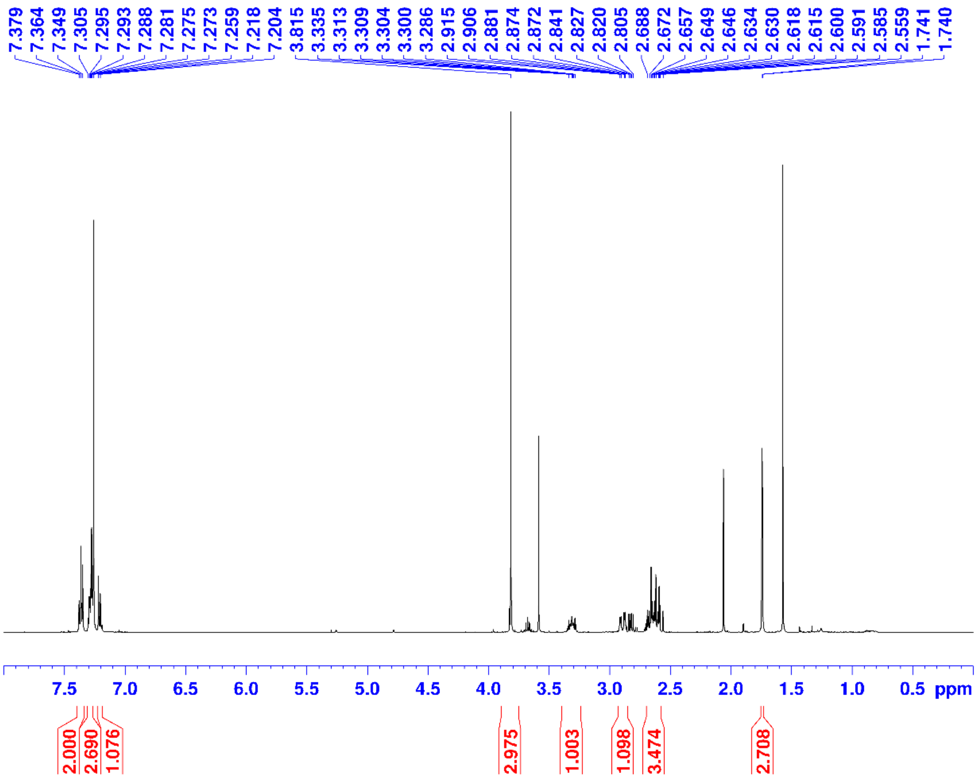

^{13}C NMR

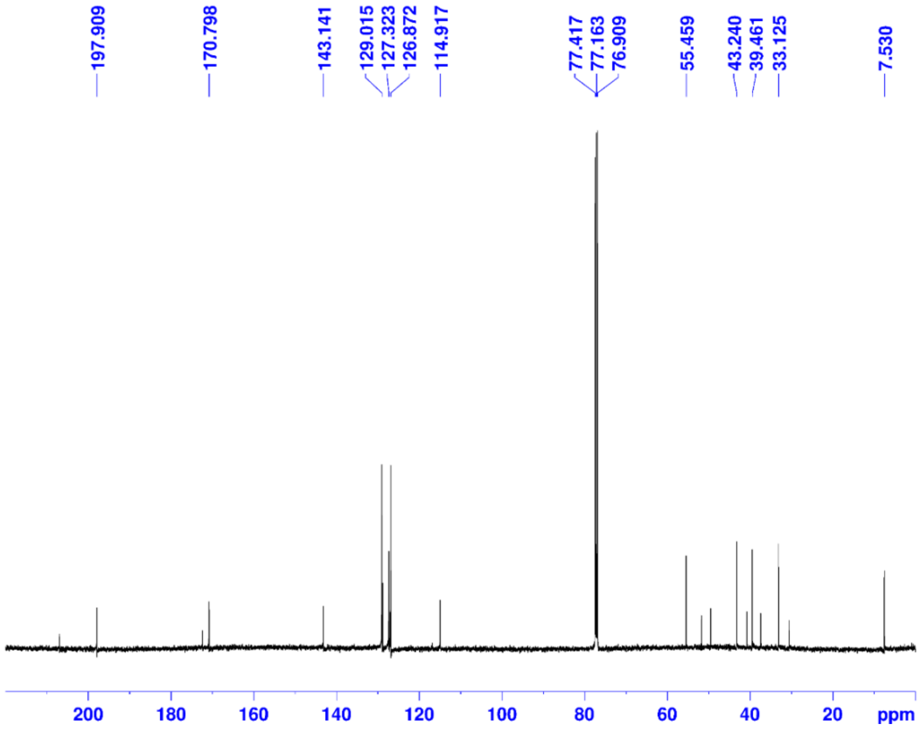

3-methoxy-5-phenylcyclohex-2-ene-1-one 8

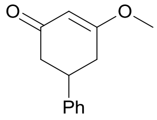

8

^{1}H NMR

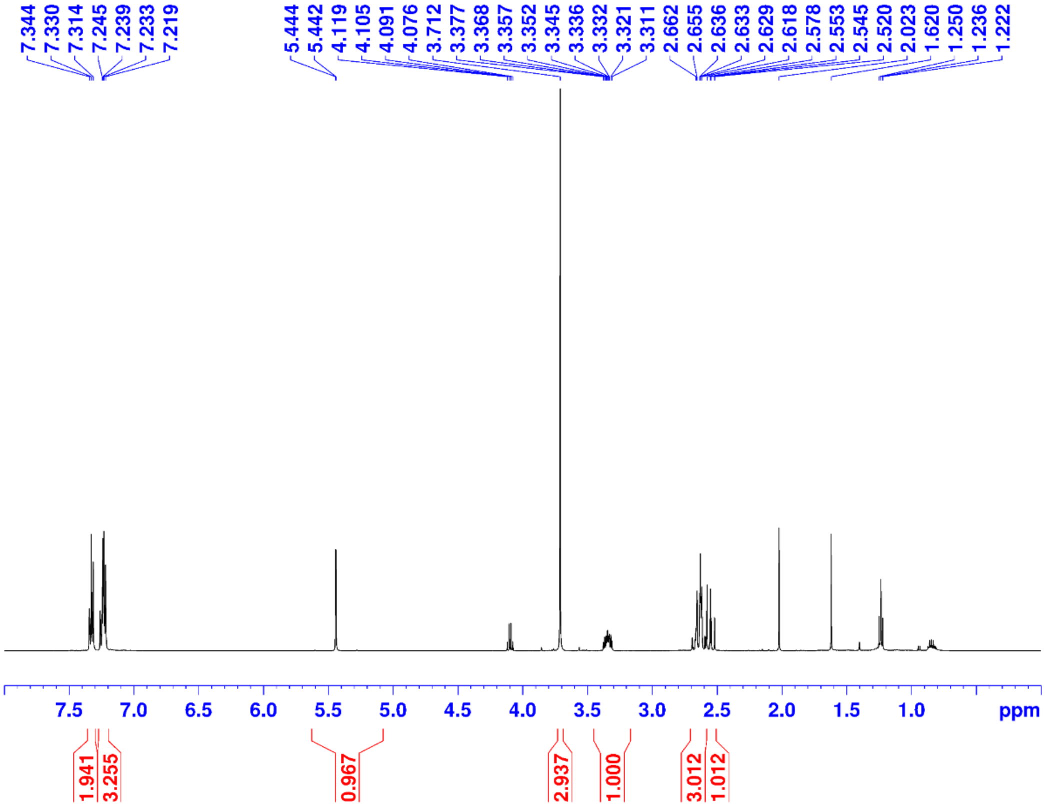

^{13}C NMR

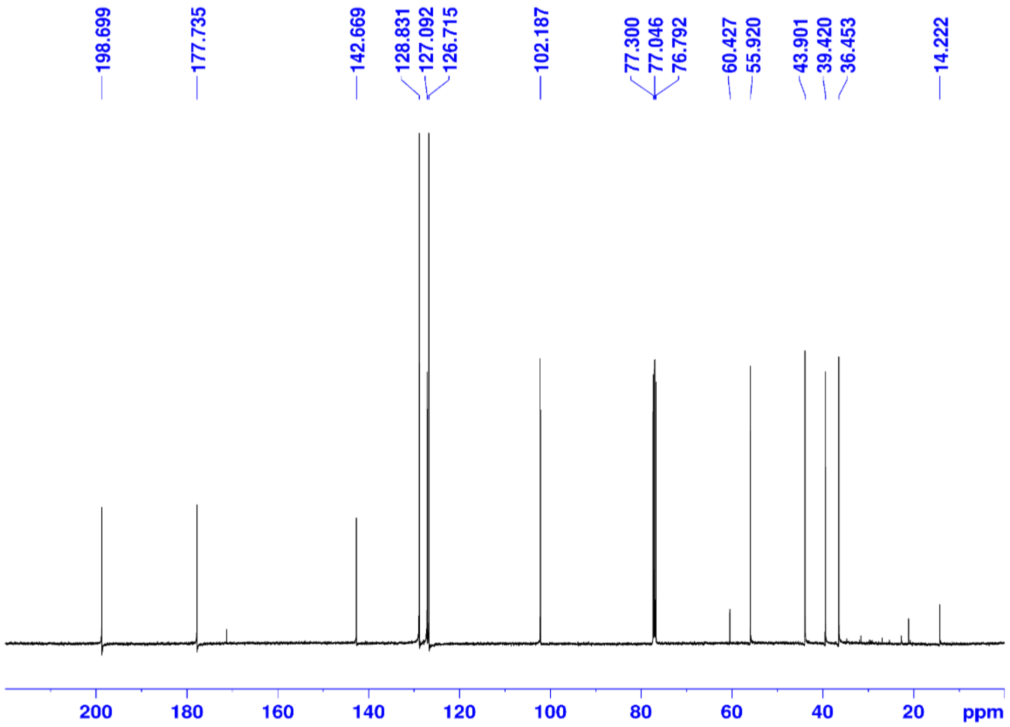

Bis-dithiane 6a

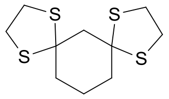

6a

^{1}H NMR

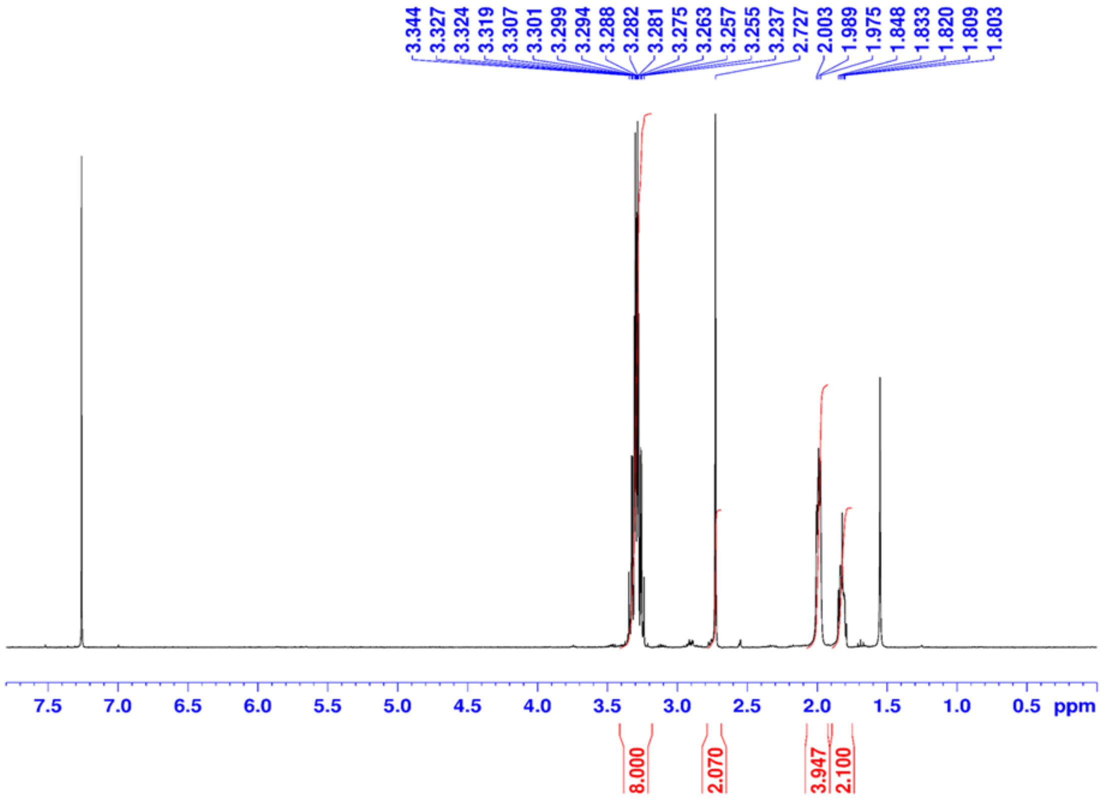

^{13}C NMR

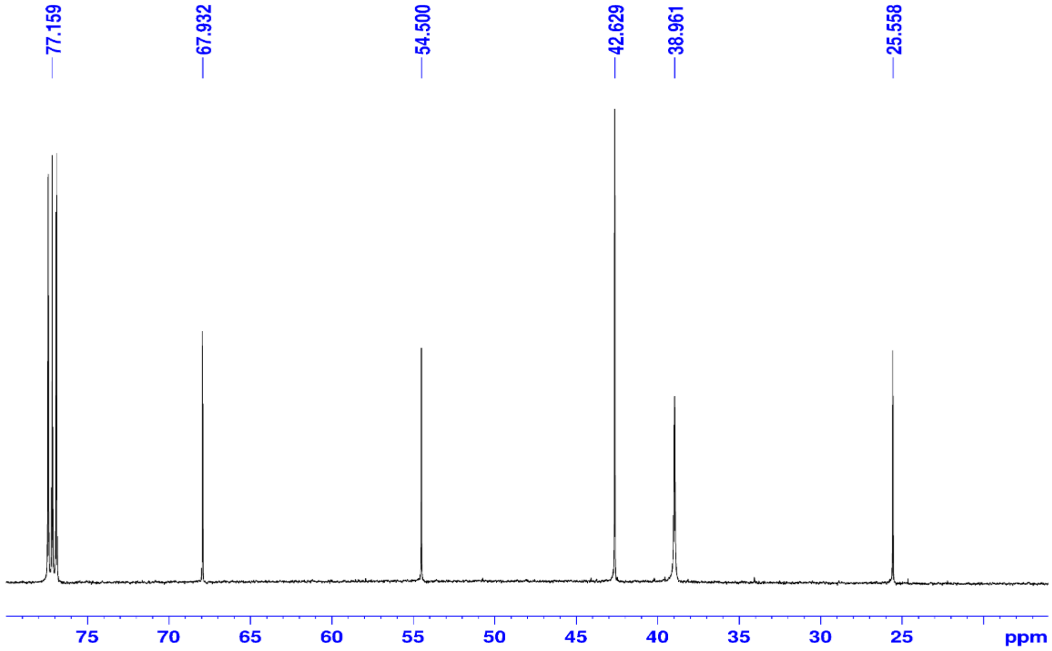

Bis-dithiane 6b

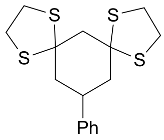

6b

^{1}H NMR

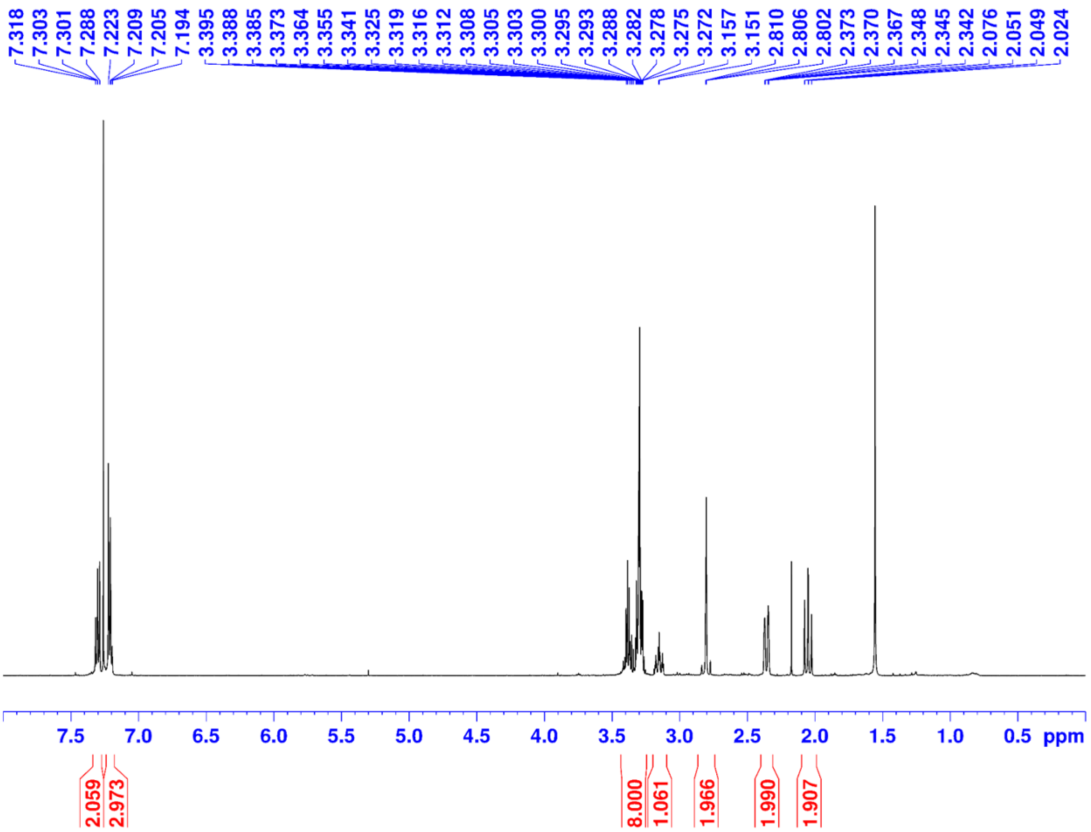

^{13}C NMR

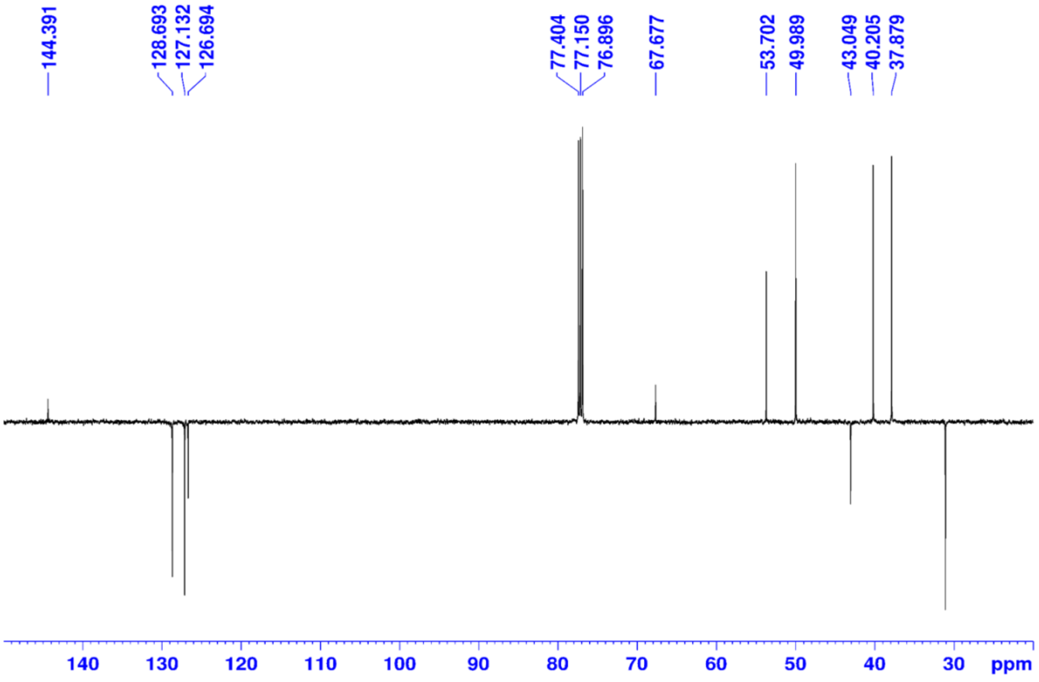

Bis-dithiane 6c

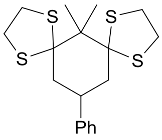

6c

^{1}H NMR

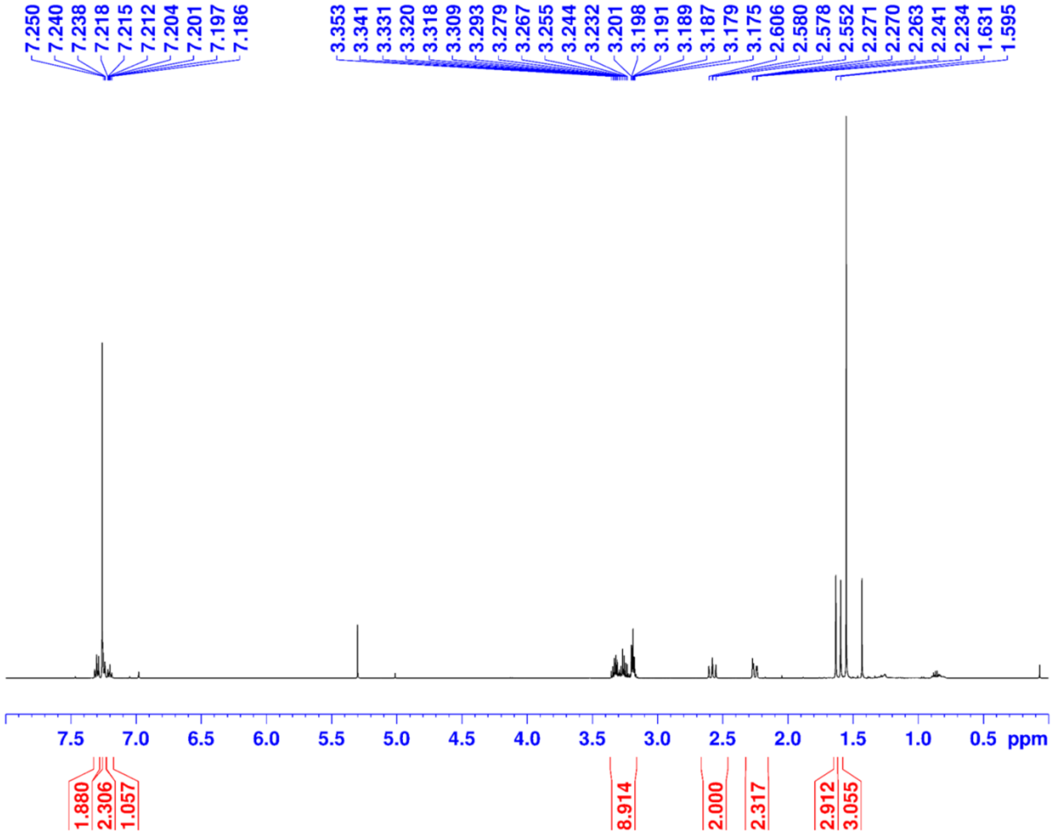

^{13}C NMR

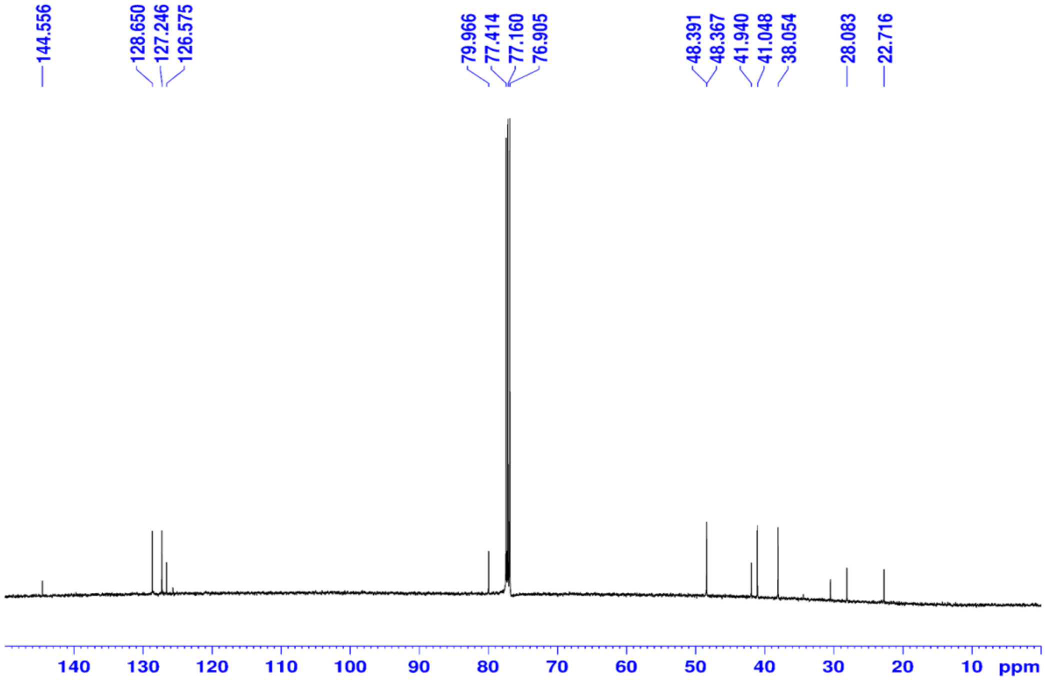

dithiane 9

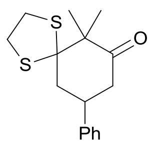

9

^{1}H NMR

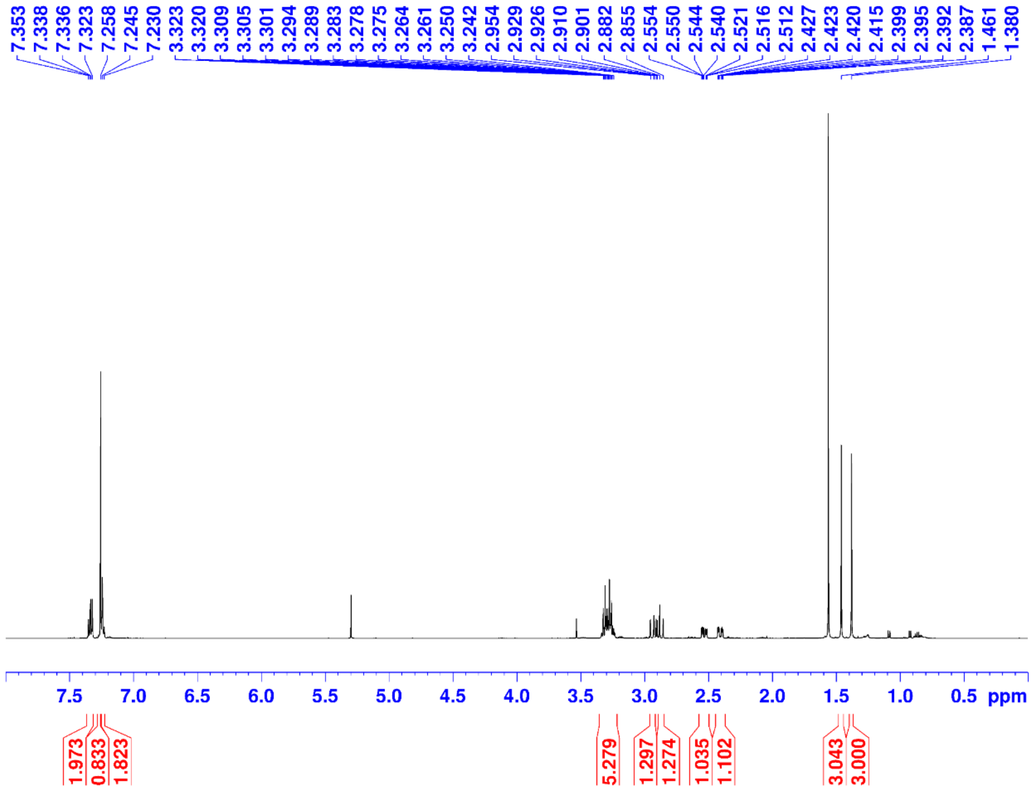

^{13}C NMR

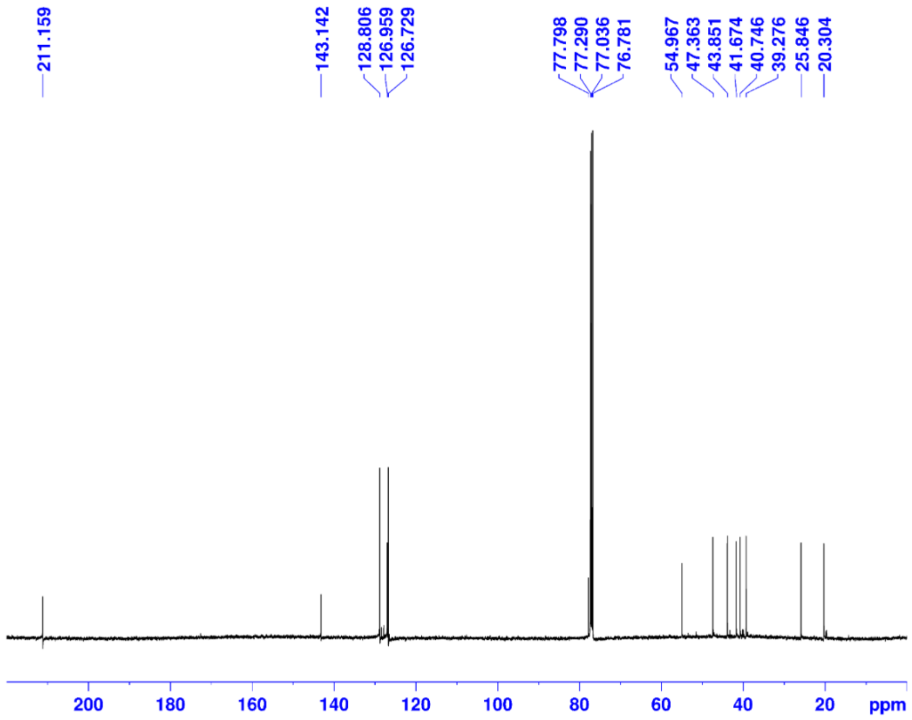

1,1,3,3-tetrafluoro-2,2-dimethyl-5-phenylcyclohexane 4c

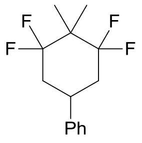

4c

^{1}H NMR

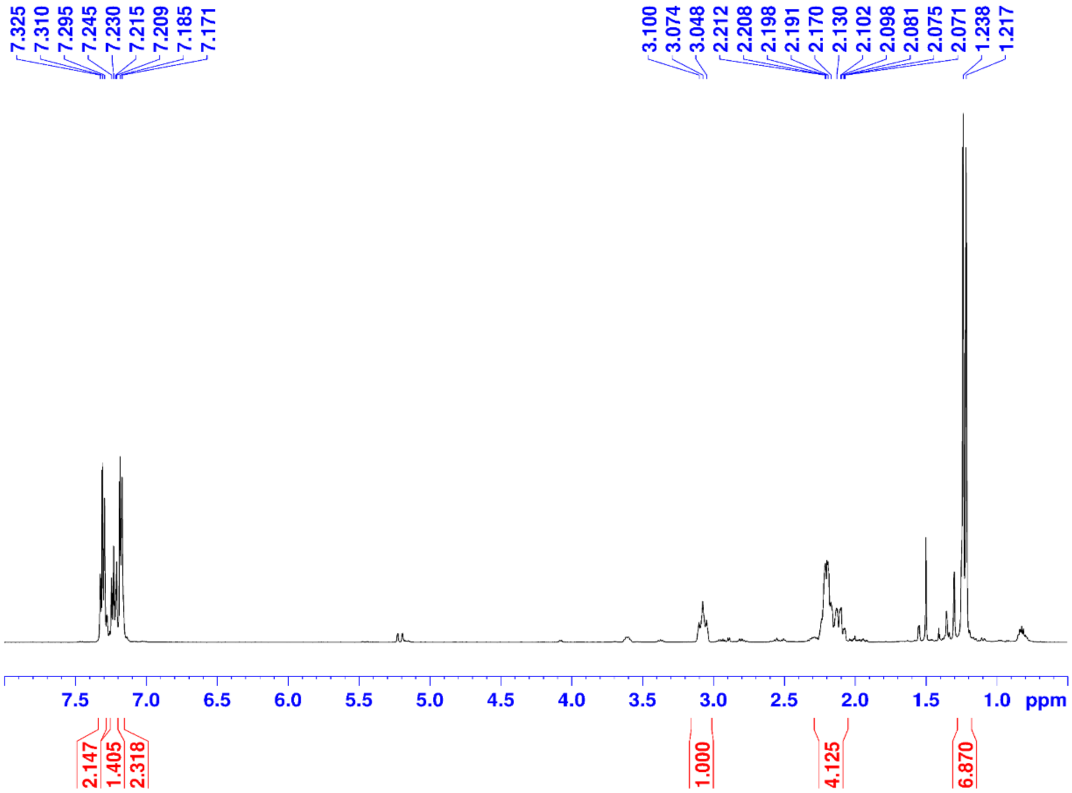

^{13}C NMR

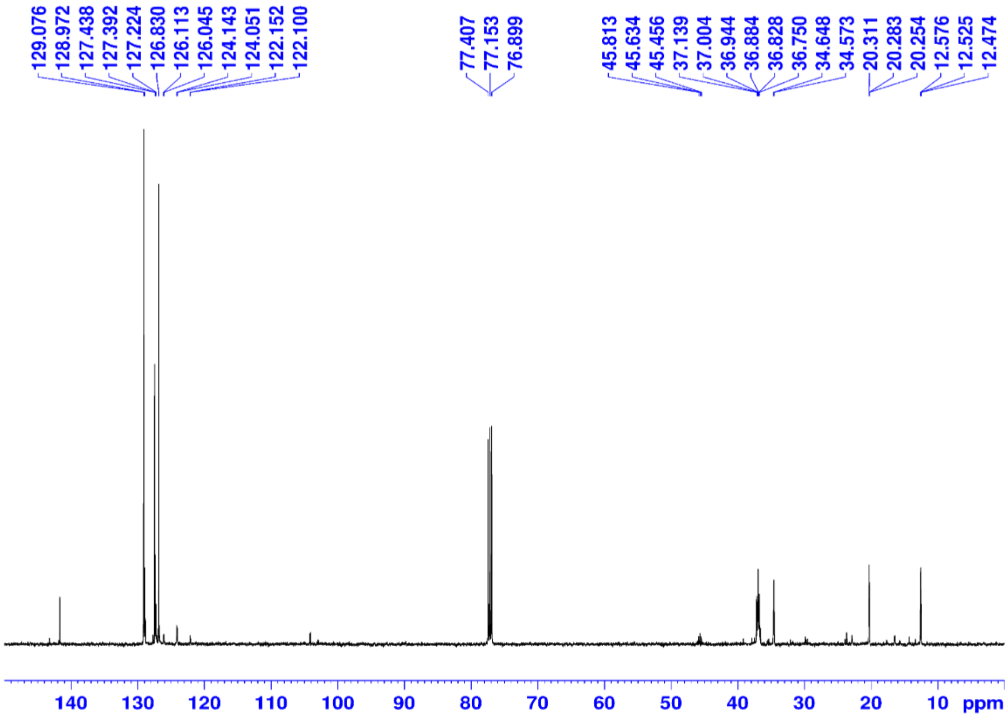

^{19}F NMR

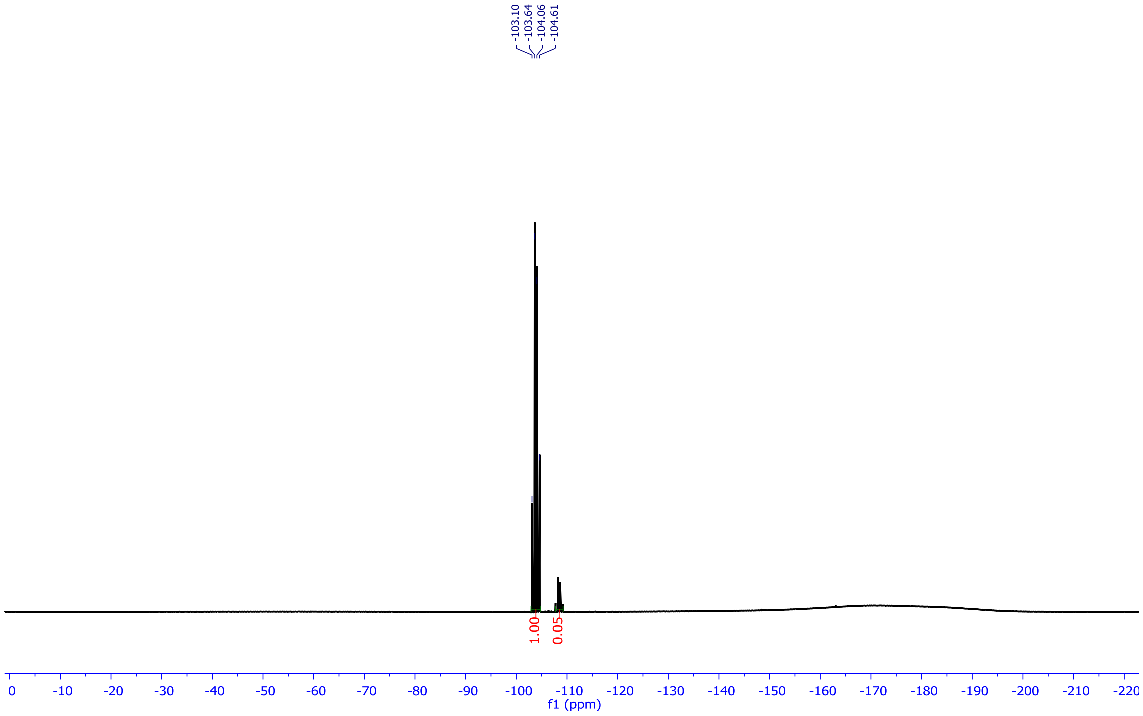

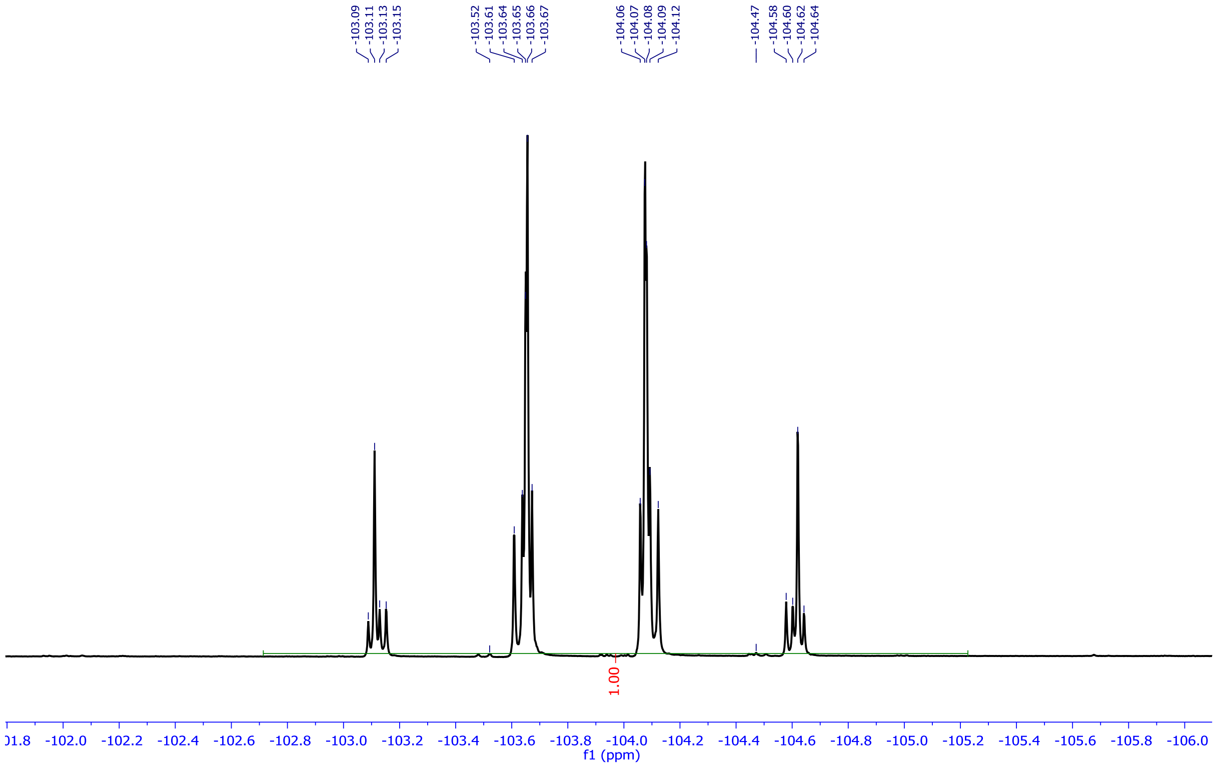
-103.09
-103.11
-103.13
-103.15
-103.52
-103.61
-103.64
-103.65
-103.66
-103.67
-104.06
-104.07
-104.08
-104.09
-104.12
-104.47
-104.58
-104.60
-104.62
-104.64
1.00
-102.0 -102.2 -102.4 -102.6 -102.8 -103.0 -103.2 -103.4 -103.6 -103.8 -104.0 -104.2 -104.4 -104.6 -104.8 -105.0 -105.2 -105.4 -105.6 -105.8 -106.0
f1 (ppm)
